# Metastases to extraocular muscles from breast cancer: case report and up-to-date review of the literature

**DOI:** 10.1186/s12885-018-5253-1

**Published:** 2019-01-08

**Authors:** Marialuisa Framarino-dei-Malatesta, Annalisa Chiarito, Federico Bianciardi, Marco Fiorelli, Azzurra Ligato, Giuseppe Naso, Irene Pecorella

**Affiliations:** 1 Department of Gynecological, Obstetrical, and Urological Sciences, University Sapienza of Rome, Policlinico Umberto I, Viale del Policlinico 155, 00161 Rome, Italy; 2 Radiotherapy - UPMC San Pietro Fatebenefratelli, Via Cassia 600, 00189 Rome, Italy; 3 Department of Neurosciences, University Sapienza of Rome, Policlinico Umberto I, Viale dell’Universita’ 30, 00185 Rome, Italy; 4 Department of Radiological, Oncological and Anatomical Pathology Sciences, University Sapienza of Rome, Policlinico Umberto I, Viale del Policlinico 155, 00161 Rome, Italy; 5 Department of Radiological, Oncological and Anatomical Pathology Sciences, University Sapienza of Rome, Viale Regina Elena 324 -, 00161 Rome, Italy; 6 Rome, Italy

**Keywords:** Extraocular muscles metastases, Breast cancer, Antiestrogen drug fulvestrant, Selective inhibitor of CDK4 /CDK6 palbociclib

## Abstract

**Background:**

Unilateral or bilateral metastases to extraocular muscles are very rare in breast cancer.

**Case presentation:**

We describe a case of inferior rectus extraocular muscle involved by ductal luminal B/Her-2 neu negative breast cancer, observed in a cohort of 580 patients. Our patient had received chemotherapy and hormonal therapy (tamoxifen for 3 years and letrozole in the following 3 years) for her primary cancer and developed an orbital metastasis while she was under aromatase inhibitor-based therapy. Diagnosis was confirmed by MRI and biopsy. Orbital radiotherapy, combined with fulvestrant, resulted in shrinking of the secondary mass. A third line hormonal therapy using palbociclib was then started. Twelve-months later, MRI showed no residual tumor mass. Currently, the patient is alive and in good general conditions after 20 months.

**Conclusions:**

Literature review yielded 57 patients with extraocular muscle metastases from breast cancer, mostly due to the invasive lobular subtype of carcinoma. In addition to the present case, only 4 other extraocular muscles metastases from invasive ductal carcinoma has been reported, pointing out to the rarity of ductal type spread to the orbit in the natural history of breast cancer. Surgery may be used as a single treatment, despite no improvement of symptoms. Radiotherapy alone or combined with chemotherapy, or with chemotherapy plus hormonal therapy are available options. Results are, however, missing or poor. The present case is the first one with complete and stable response after 20 months to radiotherapy, antiestrogen drug fulvestrant and selective inhibitor of CDK4 /CDK6 palbociclib. In this subset of patients, with unusual metastatic sites and frequent multi-organ metastatic impairment, a multidisciplinary approach is indicated in order to achieve the best therapeutic management and long-term surveillance.

## Background

Orbital metastases may be the first sign of an undiagnosed primary tumor [1], mostly invasive lobular breast cancer (ILC), as the orbit represents a rich in fat niche which may attract disseminated ILC cells [2]. On the other hand, invasive ductal breast cancer (IDC) expresses E-cadherin, which limits cell dispersion and rarely brings to orbital metastases [3]. Unilateral or bilateral metastases to extraocular muscles are very rare [4], accounting for 9% of all orbital metastases [5]. Again, breast carcinoma (BC) and melanoma are mostly the primary source, though sometimes extraocular muscles involvement is the first manifestation of other underlying malignancies, such as renal cell carcinoma [6]. We describe a case of inferior rectus extraocular muscle metastasis observed in a cohort of 580 patients with histopathologically confirmed diagnosis of BC, who attended our Oncological Day Hospital between January 1, 2011 and September 30, 2017. Additionally, we analyzed the prevalence and outcomes of these unusual metastases by literature review.

## Case presentation

**Fig. 1:**
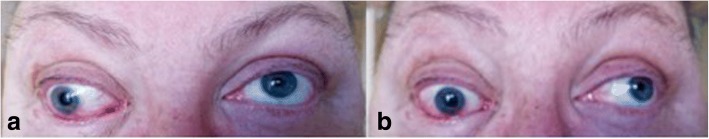
51-year-old woman with right inferior rectus extraocular muscle from BC who was diagnosed with primary ductal BC (luminal B HER-2/neu negative pT4b N3a G3) 7 years earlier, with restricted upward right eye movements (**a**). Elevation and adduction of the right eye are restricted and there is right exophthalmos (**b**)

**Fig. 2:**
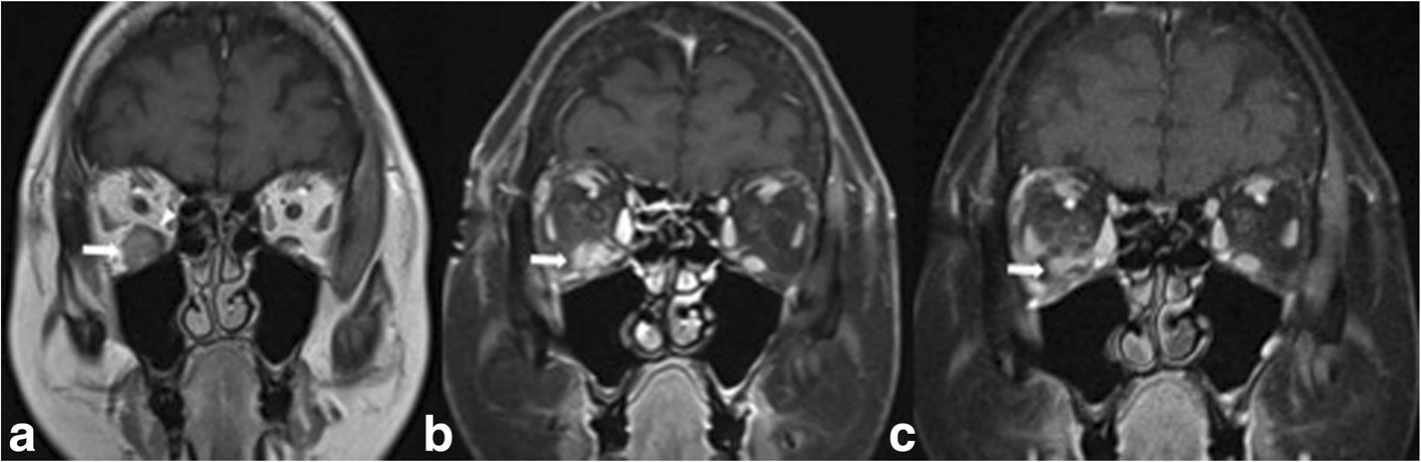
**a** T1-weighted MR image depicting the lesion at the time of first diagnosis, as a slightly hyperintense solid nodule (arrow) located on the floor of the right orbit. Part of the belly of the inferior rectus muscle appears compressed and displaced (arrowhead). Post-contrast images were not acquired in this MR examination. **b** Post-contrast fat-suppressed T1-weighted image shows the shrinkage of the lesion (arrow) 2 months after the initiation of orbital radiotherapy followed by fulvestrant-based hormonotherapy. **c** Post-contrast fat-suppressed T1-weighted image shows further shrinkage of the lesion (arrow) 17 months after the initiation of radiotherapy + hormone therapy. Palbociclib was also added between B and C, see text for details). Right inferior rectus muscle is not discernible in (**b**) and (**c**). The maximum dimension of the lesion was 14 mm in A, and decreased to 12 mm In B and to 7 mm in (**c**)

**Fig. 3:**
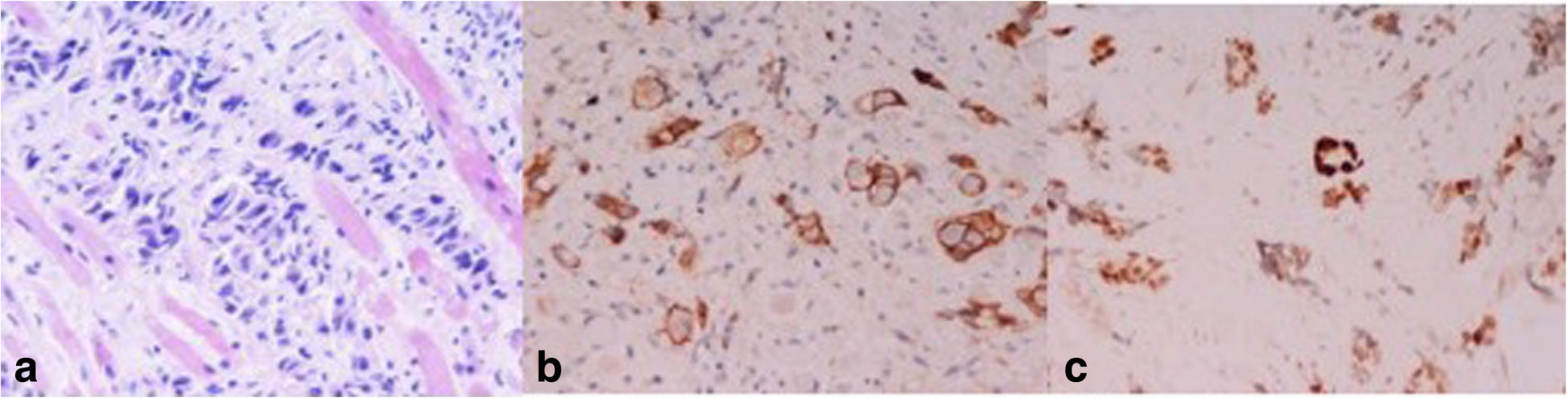
Histology shows invasion of striated muscle fibers by ductal BC (H&E, 100x) (**a**). The tumor cells are immunostained with antibodies directed against-cadherin (**b**) and ER (**c**), thus confirming their breast origin (avidin-biotin, AEC counterstain, 400 x and 250 x, respectively)

A 44 years-old woman presented with right breast pain, swelling and nipple retraction. Breast ultrasonography (US) showed an irregular hypoechoic mass (30 × 10 mm) in the right retro-areolar space; a further lesion (maximum diameter 8 mm) was detected in the right upper inner quadrant. Lymph-nodes with a maximum diameter of 25 mm were also detected in the right axilla by US. A core needle biopsy revealed a poorly differentiated (G3), estrogen receptor (ER) positive (ER+) [65%], progesterone receptor (PgR) positive (PgR+) [50%], cell proliferation antigen (Ki-67) 70%, human epidermal growth factor receptor-2 (HER-2 neu) negative, IDC. A total body Computed Tomography (CT) showed no evidence of metastatic disease. After four cycles of neoadjuvant chemotherapy with epirubicin 100 mg/m2 and taxol 175 mg/m2 every 21 days, a right “skin sparing” mastectomy and axillary lymph node dissection was performed. Immunohistochemistry confirmed G3 luminal B/HER-2 neu negative IDC subtype. Eleven out of fifteen axillary lymph nodes showed metastatic deposits (TNM: pT4b N3a M0). After chest wall radiotherapy including supra−/infraclavicular lymphatic drainage area, the patient started further eight cycles of adjuvant chemotherapy with taxol 175 mg/m2 every 21 days. Tamoxifen 20 mg daily and triptorelin 3,75 mg once a month for 3 years, and letrozole 2.5 mg daily in the following 3 years were used. Seven years after the diagnosis, while still under letrozole-based hormonal therapy, the patient displayed diplopia, blurred vision, and significantly restricted upward right eye movements (Fig. 1). Ocular acuity decreased from 7 to 2/10 in both eyes. A brain Magnetic Resonance Imaging (MRI) showed a lump involving the right inferior rectus extraocular muscle (Fig. 2a). Computed Tomography (CT) confirmed this finding without showing other sites of metastasis. A transpalpebral biopsy revealed breast cancer metastasis (ER 50%, PgR 0%, Ki67 35%, HER-2 neu negative) (Fig. 3). The patient underwent orbital Stereotactic Body Radiation Therapy (40 Gy in 5 days), combined with fulvestrant 500 mg day 1, 15, 29 and, subsequently, every 28 days. A brain CT and MRI, performed 2 months later to evaluate the treatment response, showed a shrinking of the orbital mass. (Fig. 2b). One month after MRI, following mediastinal lymph nodes enlargement, a third line therapy using palbociclib 125 mg daily for 3 weeks/month was started. Twelve-months later, MRI showed no residual tumor mass (Fig. 2c) and Positron Emission Tomography (PET) confirmed no uptake in the orbit. Despite tumor regression, the right eye sight failed to improve. Currently, the patient is alive and in good general conditions after 20 months under anti-hormonal-based therapy. Patient provided informed consensus to publish her case and release case details and other personal information and images.

## Discussion and conclusion

In this review, we searched PubMed and Medline for English literature focusing on cases of extraocular muscle metastases from BC using the following search terms: breast cancer, orbital metastases and extraocular muscle metastases. Research articles, reviews and case reports were included.

Literature review yielded 57 patients with extraocular muscle metastases from BC [7–38]. Mean age was 60 years (range 40–83). Unfortunately, several studies did not provide histological subtypying of BC, treatment details and overall survival data. Twelve patients had extraocular muscle metastases from ILC [4, 14, 20, 21, 23, 28, 31, 38], whereas in 5 patients (including our case) IDC was the primary source [29, 34–36]. Three patients had a poorly differentiated BC, four patients had an undifferentiated BC. Histology details were lacking in the remaining 34 patients. Unilateral muscle involvement was reported in 23 patients, including the present case, and bilateral involvement in 14, whereas in 21 women laterality was not specified. TC and/or MRI ascertained the diagnosis in 35 patients (including our case) and these results were subsequently confirmed at biopsy; in 12 cases autopsy or biopsy alone were conclusive. In 11 patients, biopsy was not performed. Single treatment was based on surgery in 4 patients, radiotherapy in 4 patients and hormonal therapy in 1. Combined treatments were used in 11 patients, as follows: chemo-radiotherapy in 6, chemo-radiotherapy plus hormonal therapy with selective estrogen receptor modulators (SERMs) or aromatase inhibitors (AI) in 3, radiotherapy and hormonal therapy in 2. One of the 3 patients treated by chemo-radiotherapy plus hormonal therapy even received 47.5 Gy to the extraocular muscles without any signs of improvement. Therefore, whole-body hyperthermia was administered twice, combined with vinorelbine and mitomycin. Subsequently, she underwent several sessions of local hyperthermia and superficial hyperthermia on the skin metastases, 3 times a week. She died 1 month later without any information about her condition at that time [28]. No data about treatment were provided in 35 cases, and 2 patients did not receive any treatment. For the present case, we selected radiotherapy, hormonal therapy with antiestrogen drug fulvestrant and therapy with an oral, reversible, cyclin-dependent kinases 4/6 (CDK4/CDK6) inhibitor palbociclib. In 43 patients, the clinical outcome was not specified. In 11 cases, partial improvement and in 2 instances no response after single or combined treatments were reported. Fenton et al. described another case with lateral rectus relapsing metastasis from BC, confirmed on CT scan and biopsy. Both first metastasis and relapse 2 years later were treated by orbital radiotherapy. She had been asymptomatic until her screening ophthalmic examination [24]. Our patient is the first case of full recovery following radiotherapy plus fulvestrant and palbociclib. A 4-months to 6-years overall survival was reported in 11 patients, while in 46 patients, survival data were missing. Table 1 provides clinical and histological data, treatments, outcomes and overall survival of all of the published cases included in the review.

**Table 1:** Clinical and histological data of 58 patients with extraocular muscles metastases from BC. Literature review

SOURCE	AGE (YEARS)	HISTOLOGY	INVOLVED EOMs	TREATMENTS	OUTCOMES	SURVIVAL
Wintersteiner H, [7]	58	NS	Right MR, IR	NS	NS	NS
Bedford and Daniel PD, [8]	72	NS	All EOMs Bilaterally	NT	NS	12 Days
Ashton N, [9]	58	UNDIFFERENTIATED	NS	Wide Excision	NS	2 Years and 6 Months
	49	UNDIFFERENTIATED	NS	NS	NS	6 Years
Stephen L, [10]	NS	NS	MR	Excision of the Mass	NS	NS
Thomas A, [11]	40	PD	Left LR	RT + CHT	Growth on the lateral aspect completely regressed, no eyeball movement restriction.	NS
Cuttone JM, [12]	62	NS	Left LR	NS	NS	NS
Mortada A, [13]	56	NS	Right LR	CHT + RT (40 Gy)	NS	3 Years
	55	NS	Left MR	CHT + RT (40 Gy)	NS	3 Years
	53	UNDIFFERENTIATED	Left SR	CHT + RT (40 Gy)	NS	3 Years
Weiss R, [14]	66	Lobular	Left MR	NS	NS	NS
	57	Lobular	Left SR, LR	NS	NS	NS
Citrin referred by Ashton N	NS	NS	Right LR	NS	NS	NS
Atlas SW, [15]	NS	NS	Left MR	NS	NS	NS
	NS	NS	LR Bilaterally	NS	NS	NS
Slavin ML, [16]	65	NS	Right SO	RT (30Gy)	Subjective Diplopia and Objective Ophthalmoplegia abated within three months.	NS
Goldberg RA, [17]	NS	NS	NS	NS	NS	NS
	NS	NS	NS	NS	NS	NS
Capone A, [18]	71	NS	MR and LR Bilaterally	Refused	NS	23 Months
Glazer LC, [19]	64	NS	Right MR, Left MR, LR	Tamoxifen + RT (35Gy) + CHT	No response, more advanced periocular infiltration bilaterally, metastases to multiple body sites.	NS
Van der Herjden A, [20]	47	Lobular	Right IR and IO	NS	NS	NS
Toller KK, [21]	47	Lobular	All EOMs Bilaterally	Right Anterior Orbitotomy. RT and CHT refused.	Unchanged	9 Months
Lacey B, [4]	NS	Lobular	MR and IR Bilaterally	NS	NS	NS
	NS	NS	NS	NS	NS	NS
	NS	NS	NS	NS	NS	NS
Wallace DK, [22]	79	NS	Left MR	Orbital Surgery	No diplopia in primary gaze or reading position.	NS
Lell M, [23]	63	Lobular	MR, SR and SO Bilaterally	NS	NS	NS
Fenton S, [24]	53	NS	LR	RT	Full recovery??	NS
Spitzer SG, [25]	75	PD	All EOMs Bilaterally	CHT + RT (30 Gy) + Letrozole	Improvement in ocular motility except for limited abduction.	NS
Peckham EL, [26]	52	PD	All EOMs Bilaterally	NS	NS	NS
Luneau K, [27]	45	NS	NS	NS	NS	NS
Kouvaris JR, [28]	50	Lobular	All EOMs bilaterally.	RT (47.5 Gy) + Anastrozole + CHT (vinorelbine and mitomycin) + Local Hyperthermia.	Diplopia partial amelioration.	13 Months.
Milman T, [29]	83	Ductal	Right LR, MR, SR;	Letrozole	Partial improvement in ocular motility.	NS
			Left IO, IR			
Valenzuela AA, [30]	NS	NS	NS	NS	NS	NS
	NS	NS	NS	NS	NS	NS
	NS	NS	NS	NS	NS	NS
	NS	NS	NS	NS	NS	NS
	NS	NS	NS	NS	NS	NS
	NS	NS	NS	NS	NS	NS
Murthy R, [31]	61	Lobular	Right SR, LR, IR;	RT (54 Gy) + Tamoxifen	Complete resolution at TC/PET, improvement of the ocular motility and resolution of diplopia but mild bilateral ptosis.	NS
			Left SR			
Wiggings RE, [32]	49	NS	Right IR	RT + CHT	Partial Improvement	11 months
	45	NS	Four Recti Bilaterally Right SO	RT + CHT	Partial Improvement	8 months
Magliozzi P, [33]	NS	Undifferentiated	IR	NS	NS	NS
Sharma V, [34]	43	Ductal	IR, LR	RT	Improvement	NS
Khan NA, [35]	NS	Ductal	Right LR;	Whole Brain RT	Partial Response	Alive
			Left IR, LR			
Amer NM, [36]	66	Ductal	Four Recti Unilaterally	Anastrozol + RT (35–40 Gy)	NS	NS
Pierson TM, [37]	NS	NS	NS	NS	NS	NS
	NS	NS	NS	NS	NS	NS
	NS	NS	NS	NS	NS	NS
	NS	NS	NS	NS	NS	NS
	NS	NS	NS	NS	NS	NS
	NS	NS	NS	NS	NS	NS
Homer N, [38]	47	Lobular	MR	NS	NS	NS
	63	Lobular	NS	NS	NS	NS
	67	Lobular	MR, LR	NS	NS	NS
	68	Not biopsied	Right LR, IR	NS	NS	NS
	64	Lobular	Rectus Muscle	NS	NS	NS
Current Study	51	Ductal	IR	RT (40 Gy) + Fulvestrant+Palbociclib.	Total regression of the orbital lesion at PET/TC without improvement in eyesight.	Alive

LEGEND – *EOM* ExtraOcular Muscle, *NS* Not Specified, *PD* Poorly Differentiated, *MR* Medial Rectus, *SR* Superior Rectus, *LR* Lateral Rectus, *IR* Inferior Rectus, *SO* Superior Oblique, *IO* Inferior Oblique, *NT* No Treatment, *RT* Radiotherapy, *CHT* Chemiotherapy

Metastases to extraocular muscles belong to orbital metastases, the latter with an overall incidence ranging from 1 to 13% out of all metastatic cancers [33]. In 1990, Capone et al. made a thorough review of secondary malignancies from all over the body in this unusual site [18]. Our review is, on the other hand, the first up-to-date review on extraocular muscles metastases from breast cancer. It is not fully understood why metastatic cancers are rarely seen in voluntary muscles. The constant movement of muscles could prevent the neoplastic cell deposit or produce an unsuitable chemical environment for continued neoplastic growth [9]. ILC is the more frequent histological subtype involving extraocular muscles.

We have examined the impact of these metastases in our cohort of 580 patients with histopathologically confirmed diagnosis of BC, who attended our Oncological Day Hospital between January,2011 and September,2017.

Data of 580 patients with invasive BC including patient’s age, clinical presentation and interval between diagnosis of primary cancer and metastases were extracted from our Database. We also noted Immunohistochemical analysis of the primary cancer, as well as treatment modalities and outcomes. Sixty-two of our 580 breast cancer patients (10.7%) developed metastases over a period of 6 to 168 months from diagnosis (mean value: 37 months). The patients’ age ranged from 23 to 83 years (mean value: 58 years). Clinical presentation included: breast pain (80%); partial or full swelling of the breast (70%); skin irritation (50%); nipple retraction (35%); nipple discharge (15%). HIC parameters according to Blows data [39] showed “luminal A” breast cancer subtype in 22 patients, “luminal B HER-2 neu positive” subtype in 17 patients, “luminal B HER-2 neu negative” subtype in 12 patients; “no luminal HER-2 neu positive” subtype in 6 patients and “triple negative” subtype in 5 patients. Distribution of metastatic sites in relation to histological and immunohistochemical subtypes is summarized in Table 2.

**Table 2:** Distribution of metastatic sites in relation to histological and immunohistochemical subtypes in a cohort of 62 patients

HYSTOLOGY	IHC	METASTATIC SITES^a^					TOTAL
		Lung	Liver	Bone	Brain	EOM	
DUCTAL	LUMINAL A	2	1	6	0	0	9
	LUMINAL B HER2 -	3	3	9	0	1	16
	LUMINAL B HER2 +	3	3	3	1	0	10
	NO LUMINAL HER2 +	3	2	1	2	0	8
	TRIPLE NEGATIVE	5	1	4	0	0	10
LOBULAR	LUMINAL A	1	1	3	0	0	5
	LUMINAL B HER2 -	0	0	2	0	0	2
	LUMINAL B HER2 +	0	0	1	0	0	1
	NO LUMINAL HER2 +	0	0	0	0	0	0
	TRIPLE NEGATIVE	0	0	1	0	0	1
TOTAL		17	11	30	3	1	62

*IHC* Immunohistochemistry, *EOM* Extraocular Muscles

^a^data only include the first site of metastatic disease

Bone metastases developed in 30 patients (48,3%), liver metastases in 11 (17.7%), lung metastases in 17 (27,4%) and brain metastases in 3 (4.8%). Our data are consistent with a recent retrospective study by Z. Chen et al. including 796,335 patients with BC. In our series, bone metastases from ILC were most common than IDC (91.52 and 76.04%, respectively). In the ILC group, the second most common metastatic site was the liver (19.64%), followed by the lung (13.61%) and brain (4.23%), while in the IDC group it was the lung (37.11%), followed by the liver (30.53%) and brain (8.24%) [40]. Only one case of IDC in our cohort was associated with an extraocular muscle metastasis and represents the fifth described case of such involvement, pointing out to its rarity in the natural history of IDC.

From the clinical point of view, diplopia and motility disturbances are the most common symptoms and signs of extraocular muscle secondaries. Palpable or visible mass, ocular proptosis or displacement are also common. Pain and inflammatory signs, such as swelling and chemosis, are seen in less than one quarter of patients [17]. Local pain is described in all patients with metastases involving orbital and ocular regions [20]. Histology of the lump confirms the diagnosis. The horizontal rectus muscles are more commonly affected than the vertical and oblique ones [41], as in our patient. Metastases to the extraocular muscles can be detected by US, MRI and CT scan. The latter is known to detect more than 95% of the orbital tumors and to provide clinical information about the size, quadrant location, relation to muscle cone and other structures [28]. MRI, compared to CT, offers sometimes better resolution of the mass [9], as in our case. As extraocular metastases from BC usually occur in the setting of multisystem end-stage disease, the treatment is mainly palliative [14, 25]. Surgery may be used as a single treatment [9, 10, 21, 22], despite no improvement of symptoms [12]. Radiation therapy alone (usually 30 Gy) [16, 24, 34, 35], combined with chemotherapy, [11, 13, 32] or with chemotherapy plus hormonal therapy [19, 25, 28] are available options. Single hormonal therapy has been reported [29]. AIs (anastrozole, letrozole, exemestane) are now recognized as the agents of choice for the management of post-menopausal women with steroid hormone positive metastatic BC, in whom indications for chemotherapy are not absolute [42]. Fulvestrant, a competitive estrogen receptor antagonist with comparable affinity to estradiol, is a safe and active treatment for endocrine sensitive advanced BC patients [43]. Alterations in the mechanisms governing the cell cycle induces uncontrolled cellular proliferation and CDK4/6 are recognized as potential target in ER+ BC [44]. Recently palbociclib, a selective inhibitor of CDK4 /CDK6, received a ‘Breakthrough Therapy’ designation from the US FDA in metastatic ER+ breast cancer [45]. Paloma 3 trial established a longer progression-free survival from association of palbociclib combined with fulvestrant as second-line treatment for ER+, HER2-negative advanced or metastatic BC with disease progression during or following endocrine therapy [46, 47].

According to these recent advances in metastatic BC we decided to use radiotherapy for local disease control, third-line hormonal therapy with fulvestrant that followed previous tamoxifen and letrozol treatments and selective inhibitor of CDK4 /CDK6 palbociclib for control of systemic disease, with good results so far.

The present case is the fifth IDC involving extraocular muscles, and the first with complete and stable response to radiotherapy, antiestrogen drug fulvestrant and palbociclib, in the absence of short-term side effects. We also wish to underline that these metastases are very rare and frequently are part of systemic metastatic disease from BC. In this subset of patients, rarity of the involved site and frequent multi-organ neoplastic impairment indicate a multidisciplinary approach in order to achieve the best therapeutic management. Long-term surveillance will be required to establish effective remission and any long-term side effects.

## Abbreviations

AIs: Aromatase inhibitors
BC: Breast carcinoma
CD4/6: Cyclin-dependent kinases 4/6
CHT: Chemiotherapy
CT: Computed Tomography
EOM: ExtraOcular Muscle
EOMMs: Extraocular muscles metastases
ER: Estrogen receptor
ER+: Estrogen receptor positive
HER-2 neu: Human epidermal growth factor receptor-2
HIC: Immunohistochemistry
IDC: Ductal invasive breast cancer
ILC: Lobular invasive breast cancer
IO: Inferior Oblique
IR: Inferior Rectus
Ki-67: Cell proliferation antigen
LR: Lateral Rectus
MR: Medial Rectus
MRI: Magnetic Resonance Imaging
NS: Not Specified
NT: No Treatment
PD: Poorly Differentiated
PET: Positron Emission Tomography
PgR: Progesterone receptor
PgR+: Progesterone receptor positive
RT: Radiotherapy
SERMs: Selective estrogen receptor modulators
SO: Superior Oblique
SR: Superior Rectus
US: Ultrasonography
